# Improving Color Quality of Nanowire White Light-Emitting Diodes with Mn^4+^ Doped Fluoride Nanosheets

**DOI:** 10.3390/mi12080965

**Published:** 2021-08-15

**Authors:** Thi Hong Quan Vu, Thi Tuyet Doan, Barsha Jain, Ravi Teja Velpula, Tung Cao Thanh Pham, Hieu Pham Trung Nguyen, Hoang-Duy Nguyen

**Affiliations:** 1 Institute of Chemical Technology, Vietnam Academy of Science and Technology, Ho Chi Minh City 700000, Vietnam; q.vu@intibs.pl (T.H.Q.V.); doantuyet.khtn@gmail.com (T.T.D.); pcttung@ict.vast.vn (T.C.T.P.); 2 Helen and John C. Hartmann Department of Electrical and Computer Engineering, New Jersey Institute of Technology, Newark, NJ 07102, USA; bj226@njit.edu (B.J.); rv366@njit.edu (R.T.V.); 3 Faculty of Materials Science, University of Science, Vietnam National University, Ho Chi Minh City 700000, Vietnam

**Keywords:** fluoride nanosheets, red emission K_2_TiF_6_:Mn^4+^, III-nitride, nanowire, white light-emitting diodes

## Abstract

A two-dimensional nanostructured fluoride red-emitting phosphor with an excellent quantum yield of ~91% is studied for cost-effective and high-color quality nanowire white light-emitting diodes (WLEDs). K_2_TiF_6_:Mn^4+^ phosphors are synthesized via an emulsification method using surfactants as sodium dodecyl sulphonate and oleic acid. The K_2_TiF_6_:Mn^4+^ phosphors in ultra-thin and nanosheet crystals are observed via scanning electron microscopy and high-resolution transmission electron microscopy. The surfactants are found to play a key role in inhibition of KTFM crystal growth process and stabilization of Mn^4+^ ions doping into the K_2_TiF_6_ host. The prepared phosphors exhibited intensive red emission at approximately 632 nm and excellent thermal stability in the range of 300–500 K upon 460 nm light excitation. Moreover, the K_2_TiF_6_:Mn^4+^ nanosheets were integrated on InGaN/AlGaN nanowire WLEDs for color quality study. The results show that the nanowire WLEDs with red-emitting phosphor exhibit unprecedentedly high color rendering index ~96.4, and correlated color temperature ~4450 K.

## 1. Introduction

White light-emitting diodes (WLED), composed of yellow phosphor and blue-light LED, have been replacing traditional lights such as incandescent and fluorescent lamps for general lighting because of their highlight efficiency, long lifetime and energy saving [1,2,3]. In order to improve the color rendering index (CRI) of WLED, red-emitting fluoride materials with high quantum yield area promising candidate, which is considered as a phosphor converted WLED or pc-WLED approach [4,5,6]. Alternatively, nanowire structures have been intensively studied for WLEDs [4,5,6,7,8,9,10]. In these nanowire WLEDs, intrinsic white-light emissions are generated by integrating full-color emission in a single nanowire. Nanowire WLEDs exhibit high quantum efficiency due to the reduced dislocation densities, the resultant polarization fields [5,6] and the absence of phosphor converter. However, the development of high-power red emission in LEDs still remains challenging due to the immature growth condition and large lattice mismatch resulted from high indium composition in the InGaN layers. Therefore, the utilization of red-emitting fluoride materials in nanowire LEDs offers a promising solution for achieving high power and high color quality nanowire WLEDs.

Manganese (IV) doped fluoride phosphor materials such as A_2_MF_6_:Mn^4+^ (with A = Na, K, Rb, Cs; B = Si, Ge, Ti), A_3_BF_6_:Mn^4+^ (B = Al, Ga) and BaMF_6_:Mn^4+^ (B = Si, Ti) [11] exhibit a sharp photoluminescence (PL) band, intensive red emission and low thermal quenching compared to those of rare-earth phosphor. Among the fluoride phosphors, K_2_TiF_6_:Mn^4+^ (KTFM) is noticed with dramatically strong red emission at 630 nm and high quantum yield up to 98% under UV or blue-light excitation [11,12,13,14]. Additionally, the synthesis of fluoride phosphors at room temperature is not complex, which is suitable for industry production. Owing to their special structural, electronic and optical properties, nanocrystals offer new possibilities for applications in lighting, display, energy and environmental technologies [15]. It is known that sub-micrometer-sized phosphors can facilitate a decrease in their consumption and improved resolution of phosphor screens. When the particle size becomes comparable to wavelengths of light, the optical properties of phosphor powders undergo remarkable qualitative changes [16,17,18]. Nanophosphors can be defined as nanoparticles of transparent dielectrics (hosts) doped with optically active ions (activators), so that the emission of light happens due to the electronic transitions between the levels of the impurity ions inside the bandgap of the host (characteristic luminescence) [17]. Currently, thin-KTFM red-emitting phosphor with 30–100 μm size and 3–5 μm thickness synthesized via an alcohol-assisted co-precipitation method showed a reduction of light-scattering loss. The luminous efficacy and color rendering index (CRI) of white-light emitting diodes (LEDs) using thin-KTFM has been improved remarkably [19]. In this study, we prepared K_2_TiF_6_:Mn^4+^ ultrathin and nanosheet crystals through the emulsification method using surfactants to control the thickness and size of phosphors. The effect of the surfactants on luminescent properties of KTFM phosphor is studied in detail. Moreover, a significant improvement in color quality of nanowire WLEDs integrated with KTFM nanosheets is also studied and reported.

## 2. Experimental Section

### 2.1. Chemicals

Sodium dodecyl sulphonate (SDS), oleic acid (OA), potassium permanganate (KMnO_4_ 99%)*,* potassium fluoride (KF.2H_2_O 99%), hydrofluoric acid (HF 40%), titanium(IV) isopropoxide (Ti-iso 97%), isopropanol (99%), acetone (98%) and hydrogen peroxide solution (H_2_O_2_ 30%): All chemicals were of standard grade and used as received without further purification.

### 2.2. Synthesis of K_2_TiF_6_:Mn^4+^

The K_2_TiF_6_:Mn^4+^(KTFM) nanoparticles were synthesized by an emulsification method. A mixture of 0.13 g KMnO_4_, 3.2 g KF.2H_2_O and 0.05 g SDS surfactant was dissolved in 20 mL HF 40% solution (Solution A). Solution B composed of 5 mL isopropanol, 0.5 mL Ti-iso and OA (with various weight ratios of OA/SDS ~ 0.0, 2.0, 6.0 and 10.0). A solution of 10 μL H_2_O_2_ and 5 mL isopropanol was dropped into a mixture of A and B solutions under vigorous stirring at 3 °C until the violet solution turned to deep yellow. The yellow precipitates were isolated and washed with HF acid 20% and then with acetone for several times. Finally, the sample was dried at 50 °C for 2 h in oven vacuum.

### 2.3. Fabrication of K_2_TiF_6_:Mn^4+^ on Nanowire WLED

The InGaN/AlGaN nanowire WLED heterostructures were grown by a Veeco GEN II molecular beam epitaxy (MBE) system under nitrogen-rich condition. The device structure consists of ten couples of 3 nm InGaN/3 nm AlGaN quantum dot (QD) active region sandwiched in between a ~200 nm GaN:Si template and ~200 nm GaN:Mg layer. The emission spectrum of the nanowire WLEDs is optimized so that red/green/blue emissions are generated from a single nanowire. The color emission of the nanowire WLEDs can be optimized by controlling the indium composition in the InGaN active region. The nanowire LED devices are then fabricated from nanowire LEDs on Si wafer using the following procedure. The nanowires are coated with polyimide by spin-coating, then oxygen-plasma dry etching to expose the nanowires’ tips. Anode electrode (*p*-contact) is fabricated by evaporating thin nickel (Ni), gold (Au) and indium tin oxide (ITO) layers on top of the nanowires, respectively. Subsequently, thick Ni and Au layers are covered on the top of the ITO layer. The back side of the silicon wafer is deposited with titanium (Ti) and then Au layers for cathode electrode (*n*-contact). The epitaxial growth and device fabrication of such nanowire WLED structures are reported elsewhere [6,7,20,21]. An isopropanol suspension of 6.0wt% K_2_TiF_6_:Mn^4+^ was then coated onto the surface of the 100×100 µm^2^ nanowire WLED via a spin coating method.

### 2.4. Characterization of Materials

The solid products were characterized by using X-ray powder diffraction (X’Pert Pro Panalytical X-ray using Cu-Kα radiation, λ = 1.54056 Å and a graphite monochromator operating at 40 kV and 30 mA between 10° and 70° at a scanning rate of 0.026 °/s). The morphologies of phosphors are observed through a scanning electron microscope (SEM, JEOL, JSM-6700F) and high-resolution transmission electron microscopy (HRTEM, JEOL, JEM-2100). The photoluminescence excitation (PLE) and photoluminescence (PL) spectra were measured using an F-7000 FL spectrophotometer equipped with a 150-W xenon lamp at room temperature. Steady-state luminescence spectra were excited with the wavelength of 460 nm. For the temperature-dependent measurements, the samples were placed in a small platinum hold with its temperature controlled by a Linkam THMS600 heating/freezing stage (Linkam Scientific Instruments Ltd., Tadworth, UK). Light was radiated by a Hamamatsu R928 photo-multiplier tube. The quantum yield was measured using the Hamamatsu PMA-12 spectrophotometer equipped with an integrating sphere. The electroluminescence (EL) of the LED devices was collected by an optical fiber and analyzed using an USB2000 Ocean Optics spectrometer at room temperature.

## 3. Results and Discussion

**Figure 1:**
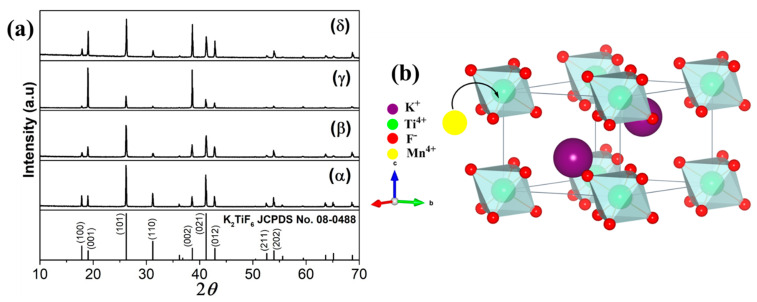
(**a**) XRD patterns of KTFM prepared with various OA/SDS weight ratios of (α) 0.0, (β) 2.0, (γ) 6.0 and (δ) 10.0. (**b**) Picture of K_2_TiF_6_ structure viewed along the [100] direction.

X-ray diffraction patterns of the prepared KTFM samples are shown in Figure 1a. All diffracted peaks can be indexed to the space group $D_{3d}^{3}$-*P*-3*m1* of the hexagonal structure K_2_TiF_6_ (JCPDS No. 08-0488). The characteristic diffraction peaks of manganese oxide were no observed in the XRD of KTFM samples. Crystal structure of KTFM is depicted in Figure 1b. Each Ti^4+^ ion is surrounded by 6 F^−^ ions, resulting in formation of [TiF_6_]^2–^ octahedral. K^+^ ions were coordinated by 12 F^−^ ions to form [KF_12_]^11−^ polyhedron and located among octahedral to balance the structure.

**Figure 2:**
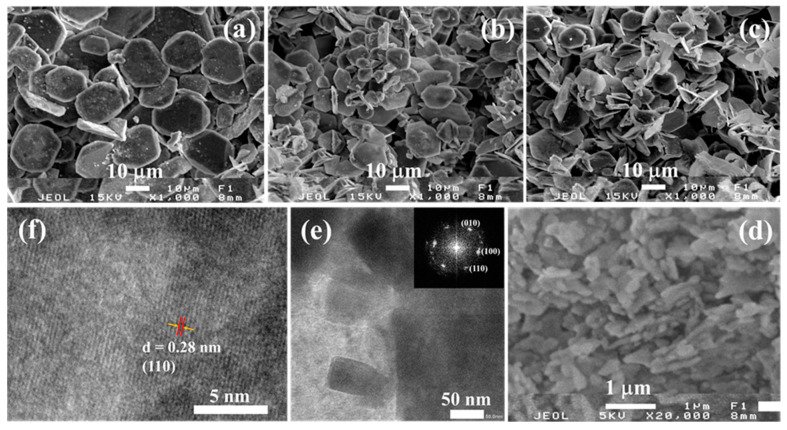
SEM images of KTFM prepared with different OA/SDS weight ratios of (**a**) 0.0, (**b**) 2.0, (**c**) 6.0 and (**d**) ~10.0. (**e**) TEM image and (**f**) HRTEM image of KTFM nanosheets. Inserted picture shows SAED pattern of KTFM nanosheets.

The SEM images of the KTFM samples are shown in Figure 2. In the absence of surfactant, the KTFM sample exhibited a hexagonal shape with average size and thickness of ~35 μm and ~3 μm, respectively (Figure 2a). A remarkable decrease in size of ~15 μm and thickness of ~500 nm is observed for KTFM samples prepared using OA and SDS with the weight ratio ~2.0 and ~6.0 (Figure 2b,c).The KTFM nanosheets with size in the range of 200–300 nm and thickness of 70–90 nm were obtained as increasing the OA/SDS ratio up to ~10.0 (Figure 2d). It is suggested that the SDS reduced interface surface tension of the mixture solutions in synthesis process and OA was active as ligands covering onto KTFM nuclei surface and inhibited further crystal growth [22]. The crystal passivation effect was not obvious using only OA or SDS. The TEM image and the selected area electron diffraction (SAED) pattern indicate that the KTFM nanosheets are single crystals (Figure 2e and the inserted picture). The high-resolution TEM (HRTEM) image with the interplanar spacing of 0.28 nm corresponding to the (110) crystal planes of hexagonal K_2_TiF_6_, presented in Figure 2f, reflects the crystalline nature of the KTFM nanosheets. 

**Figure 3:**
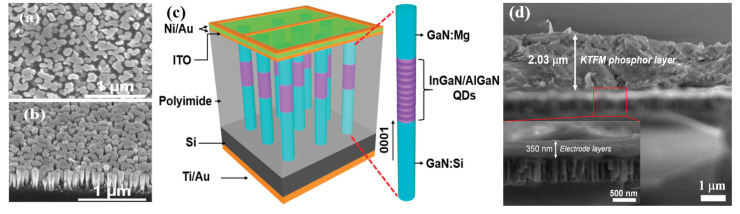
SEM images of the (**a**) top-view and (**b**) 45° tilted angle of a typical InGaN/AlGaN nanowire WLED. (**c**) Schematic illustration of the nanowire-WLED device with anode and cathode electrodes. The schematic structure of a single nanowire WLED is also presented. (**d**) SEM images of the nanowire WLEDs with KTFM nanophosphor layer. The inserted picture shows the electrode layer on the top of the nanowire WLED.

Figure 3a,b presents the top-view and 45° tilted angle SEM images of a typical InGaN/AlGaN nanowire WLED sample grown by MBE. The nanowires exhibit quite uniform wire diameters and lengths. Figure 3c illustrates the schematic structure of a single nanowire WLED that includes GaN:Si, InGaN/AlGaN active region and GaN:Mg layers. The fabricated nanowire LED device is illustrated in Figure 3c showing a schematic structure nanowire-LED device with anode and cathode electrodes and can be found in our previous publication [9]. The SEM images of the coated nanowire LEDs with KTFM red nanophosphor are shown in Figure 3d. The top electrode, nanowire LED on Si, and KTFM are clearly presented. The thickness of the KTFM layer on the LED is estimated to be ~2 µm. The *p*-contact electrode layer with thickness ~350 nm, which includes Ni/Au and ITO, is shown in inserted picture of Figure 3d.

**Figure 4:**
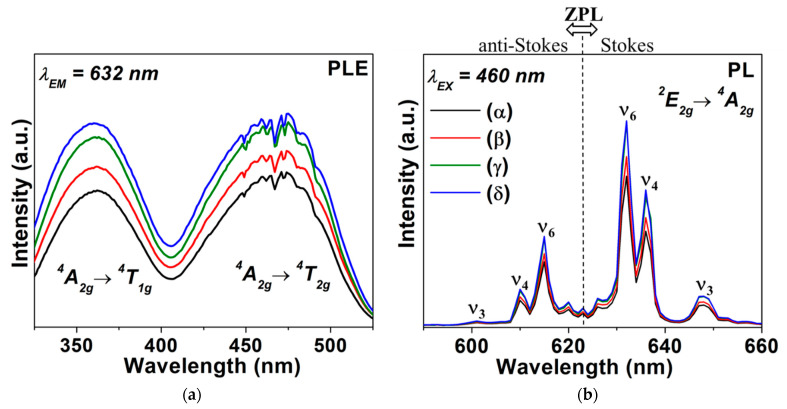
(**a**) PLE spectra and (**b**) PL spectra of KTFM prepared with various OA/SDS ratios of (α) 0.0, (β) 2.0, (γ) 6.0 and (δ) 10.0.

Figure 4a illustrates the photoluminescence excitation (PLE) spectra of prepared K_2_TiF_6_:Mn^4+^ samples at room temperature. The excitation bands of the Mn^4+^ doped K_2_TiF_6_ nanocrystals assigned to the ^4^*A*_2_ → ^4^*T*_1_ and ^4^*A*_2_ → ^4^*T*_2_ transitions are located at the 330–400 nm and 430–500 nm regions with the maxima at 350 nm and 460 nm, respectively. The emission at 632 nm (*λ*_em_ = 632 nm) was monitored. The sharp red emissions in the range of 600–680 nm, originated from the spin-forbidden ^2^*E*_g_ → ^4^*A*_2g_ transitions of Mn^4+^ in octahedral crystal-field, are presented in photoluminescence (PL) spectra (Figure 4b). The peaks at ~601, 610, 615, 622, 632, 636 and 648 nm are due to transitions of the *ν*_3_(*t*_1u_), *ν*_4_(*t*_1u_), *ν*_6_(*t*_2u_), zero phonon line (ZPL), *ν*_6_(*t*_2u_), *ν*_4_(*t*_1u_) and *v*_3_(*t*_1u_) vibronic modes, respectively, under blue-light excitation (λ_ex_ = 460 nm). The shape of the spectrum is characteristic of Mn^4+^ doped A_2_XF_6_ materials [23]. The emission intensity of KTFM samples increased gradually following the weight ratios of OA/SDS ~0.0, 2.0, 6.0 and 10.0. The KTFM nanosheet exhibited an excellent quantum yield (QY) of ~91.1%, which is much higher than that of KTFM without using surfactants ~63.1% and close to that of the KTFM micro-phosphor prepared via the cation exchange method ~93% [12,24].

**Figure 5:**
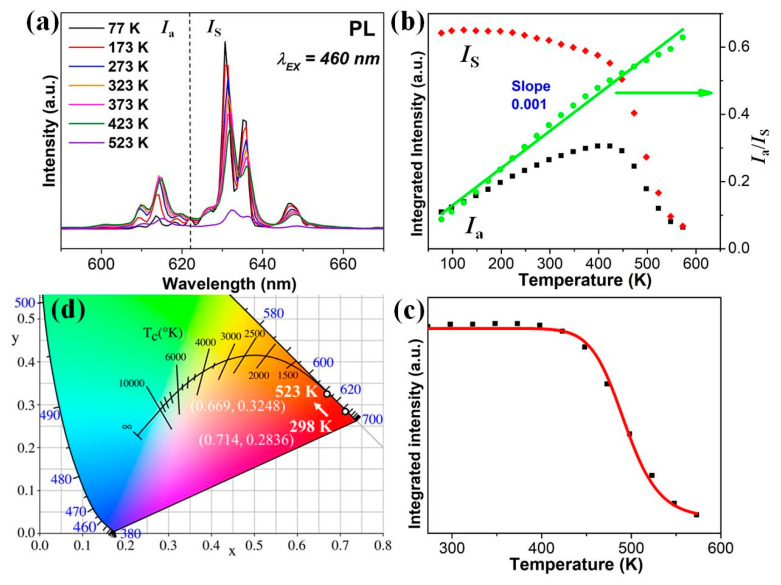
(**a**) Temperature-dependent PL spectra of the KTFM nanosheets in the range of 77 K–523K. (**b**)The integrated intensity of anti-Stokes and Stokes emission lines. (**c**) The relative red PL intensity as a function of temperature (273 K–573 K). The solid line represents the fitting result with the expression *I*_T_/*I*_0_ = [1 + *D* exp(−*E*_a_/k_B_T)]^−1^. (**d**) CIE chromaticity coordinates (x, y) shift from 298 K to 523 K of KTFM nanosheets.

Figure 5a shows the emission spectra of K_2_TiF_6_:Mn^4+^ nanosheets in the temperature range of 77 K–573 K upon 460 nm excitation. The emission peaks became broader and a slight red shift occurred within creasing temperature. The integrated intensity of anti-Stokes emission lines (*I*_a_) gradually increased in temperature range of 77–450 K, whereas that of Stokes emission lines (*I*_S_) slight decreased. Subsequently, both emission lines degraded with further increasing temperature (>450 K), which demonstrated a good color quality of KTFM nanosheets at high temperature. The ratio of *I*_a_/*I*_s_, increasing from ~0.09 at 77 K to ~0.578 at 523 K, shows a linear temperature dependence of red emission, as depicted in Figure 5b. The temperature-dependent performance of the integrated PL intensity presented in Figure 5c denotes considerable stability of KTFM nanosheets within the temperature range of 273–573 K. The integrated PL intensity of the sample started to drop at 423 K, and the quenching temperature *T*_1/2_ reached 500 K; this thermal stability is as good as that of the KTFM thin phosphor [19]. The decrease in the integrated PL intensity is attributed to the temperature-induced carriers escaping from the emission centers leading to a nonradiative recombination. Non-radiative transition probability increased with temperature, and the integrated PL intensity exhibited thermal quenching, which can be described by using the follow in gequation:

$$I_{\left(T\right)}=\frac{I_{0}}{1+A.e^{\frac{-Ea}{k_{B}T}}\;}$$

where *I*_0_ is the initial intensity at 298 K, *I*_(*T*)_ is the emission intensity at temperature *T*, *A* is a constant, *k* is the Boltzmann constant and *E*_a_ is the thermal activation energy. The *E*_a_ value obtained for KTFM nanosheets ~1.09 eV is considerably higher than that of the KTFM micro-phosphor (~0.34 eV) [25]. Figure 5d presents the changes in the International Commission on Illumination (CIE) chromaticity coordinates of the KTFM nanosheets at different temperatures. At 298 K, the CIE coordinates of KTFM nanosheets was (0.7140, 0.2836) showing a deep-red emission compared to the currently reported Mn^4+^ doped fluorides with chromaticity coordinate (0.660–0.688, 0.305–0.337) [26]. When temperature increased from 298 K to 523 K, the CIE coordinates slightly shifted in the red region from (0.7140, 0.2836) to (0.6690, 0.3248).

**Figure 6:**
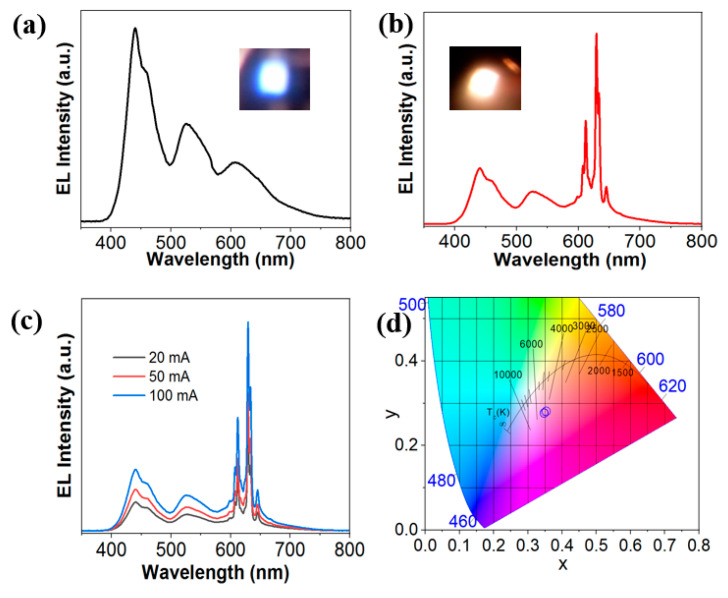
(**a**) Electroluminescence spectrum of the nanowire WLED without nanosheet KTFM red phosphor. The inset is the optical image of white-light emission from the nanowire WLED. (**b**) Electroluminescence spectrum of the nanowire pc-WLED showing clear red emission from the KTFM nanosheets. The inserted pictures show bright warm-white light emissions of the nanowire pc-WLEDs. (**c**) Electroluminescence spectra of the nanowire pc-WLED under injection current from 20–100 mA. (**d**) The chromaticity coordinate in Commission Internationale de IʹÉclairage (CIE) 1931 color spaces of the nanowire pc-WLED.

The EL spectra of the nanowire WLED without using nanosheet KTFM red phosphor and the nanowire pc-WLEDs with KTFM red phosphor are presented in Figure 6. As illustrated in Figure 6a, three dominant peaks at ~440 nm, 525 nm and 610 nm are clearly presented corresponding to the blue, green and red emissions from the InGaN/AlGaN active regions. The nanowire WLED exhibits strong white-light emission with CRI of ~85. The inset figure shows the optical image of the nanowire WLED under 20 mA injection current. Figure 6b reveals the EL spectrum of the nanowire pc-WLED with a driving current of 20 mA. The sharp emission lines of Mn^4+^ in the K_2_TiF_6_ lattice were observed in the EL spectrum. The excellent CRI values, i.e., *R*_a_ ~96.4 and the corresponding corelated color temperature (CCT) ~4450 K were recorded. The bright warm white-light emissions of nanowire pc-WLEDs are shown in the inserted picture of Figure 6b. Moreover, the nanowire pc-WLED exhibits superior color quality with strong and stable white-light emission, which was recorded for injection current in the range of 20–100 mA, as shown in Figure 6c. The chromaticity coordinates are almost invariant, which are x ~ 0.3476–0.3481 and y ~ 0.2778–0.2782, as illustrated in Figure 6d. The related CCT shows a negligible variation which is in the range of 4249–4450 K. The stable white-light emission from the nanowire pc-WLEDs is attributed to the reduced quantum confined Stark effect in the high quality InGaN/AlGaN nanowire heterostructure combining with the high efficiency and thermal stability nanosheet KTFM red phosphor. The results suggest that KTFM nanosheet is a promising candidate for improving the color reproducibility of micro-WLEDs and current commercial WLEDs.

## 4. Conclusions

In summary, high efficiency K_2_TiF_6_:Mn^4+^ nanosheets were synthesized via the emulsification method using sodium dodecyl sulphonate and oleic acid surfactants. The prepared K_2_TiF_6_:Mn^4+^ exhibited strong and stable red emission with high quantum yield ~91%. Moreover, the K_2_TiF_6_:Mn^4+^ nanosheets shows a high color quality and low thermal quenching with the relative luminescent intensity of ~100% at 423 K. We further demonstrated that, with an integration of such a KTFM nanosheet, the InGaN/AlGaN nanowire WLEDs could achieve an unprecedentedly high CRI ~96.4 and CCT ~4450 K, as recorded. The present study demonstrates that KTFM nanosheet is a promising red component for mini/micro display technologies.
